# Association of Gut Microbial Dysbiosis and Hypertension: A Systematic Review

**DOI:** 10.7759/cureus.29927

**Published:** 2022-10-04

**Authors:** Shaili S Naik, Shivana Ramphall, Swarnima Rijal, Vishakh Prakash, Heba Ekladios, Jiya Mulayamkuzhiyil Saju, Naishal Mandal, Nang I Kham, Rabia Shahid, Sathish Venugopal

**Affiliations:** 1 Internal Medicine, California Institute of Behavioral Neurosciences & Psychology, Fairfield, USA; 2 Internal Medicine, Surat Municipal Institute of Medical Education and Research (SMIMER) Hospital and Medical College, Surat, IND; 3 Research, American University of Antigua, Osbourn, ATG; 4 Internal Medicine, Government Medical College Kozhikode, Kozhikode, IND; 5 Department of Psychiatry, California Institute of Behavioral Neurosciences & Psychology, Fairfield, USA; 6 Internal Medicine, Sree Narayana Institute of Medical Sciences, Ernakulam, IND; 7 General Surgery, Government Medical College, Thiruvananthapuram, Trivandrum, IND

**Keywords:** high blood pressure, intestinal symbiosis, gut microbiome, hypertension, gut dysbiosis

## Abstract

Hypertension (HTN) is one of the most prevalent and dangerous cardiovascular diseases worldwide. Recently, its direct or indirect association with gut dysbiosis has been an interest of study for many. It also includes the metabolomic and functional gene changes in hypertensives compared with healthy individuals. This systematic review aims to study quantitative and qualitative interactions between the two and re-defining the heart-gut axis. We have strictly followed the *Preferred Reporting Items for Systematic Reviews and Meta-Analyses* (PRISMA), 2020, guidelines. We conducted an in-depth search of databases such as PubMed, PubMed Central (PMC), Medline, and ScienceDirect to find relevant studies for our topic of interest. After the final quality check, we included eight articles in the systematic review. A significant difference in richness and diversity in gut microbiota was observed in hypertensive patients compared with healthy controls. There was an increased abundance of many bacteria such as *Catabacter*, *Robinsoleilla*, *Serratia*, Enterobacteriaceae, *Ruminococcus torques*, *Parasutterella*, *Escherichia*, *Shigella*, and *Klebsiella*, while a decreased abundance of *Sporobacter*, *Roseburia hominis*, *Romboutsia* spp., and *Roseburia*. Alteration of the composition also varied based on diet, age, ethnicity, and severity of HTN. Short-chain fatty acids (SCFAs)-producing bacteria are found to be on the lower side in hypertensives owing to the protective property of SCFAs against inflammation, especially butyric acid. From the perspective of metabolomic changes, harmful metabolites for cardiovascular health such as intestinal fatty acid binding protein (I-FABP), lipopolysaccharides (LPSs), zonulin, sphingomyelins, acylcarnitines, and trimethylamine *N*-oxide (TMAO) were found to be increased in hypertensives. Changes in these biomarkers further establish the relation between gut epithelial health and high blood pressure (BP). Participants affected by diseases have an overall lower rate of acquiring new genes, which results in a low richness of genes in them compared with healthy individuals. There is increased expression of the choline utilization (*cutC*) gene and reduced expression of genes associated with biosynthesis and transport of amino acids in high-BP participants. The unique changes in the composition of the microbiota, functional changes in genes, and metabolome collectively help for a better understanding of the pathogenesis of HTN and also suggest the gut as a promising new therapeutic target for HTN. To establish a further causal relationship between the two, more research is required.

## Introduction and background

Hypertension (HTN), a major health concern worldwide, is one of the major risk factors for cardiovascular diseases. The American Heart Association statistics for 2009-2012 show approximately 80 million adults aged over 20 years have HTN. In 10 years (2003 and 2013), high blood pressure (BP) accounts for 34% of deaths [1]. Even with aggressive management by antihypertensive drugs and lifestyle modifications, only one out of five hypertensive patients manage to control BP adequately. For many researchers, it has become a center of attention mainly because its prevention can aid in reducing the number of kidney diseases and stroke in addition to cardiovascular complications [2]. As we know, lifestyle and environmental factors also contribute to the pathogenesis of HTN, such as daily salt consumption in the diet, alcohol intake, and exercise routine [3]. Recently, the gut microbiome has been newly identified as a risk factor for HTN [4,5].

Gut microbiome and gut dysbiosis are two important concepts. The human body is a host to a variety of microbial species, known as the gut microbiome, which outnumbers the cells of our body [6-8]. They are present in the oral, vaginal, gastrointestinal, and skin communities [6,7,9-11]. Their contribution ranges from playing a major role in nutrition to susceptibility to disease [11]. The imbalance in bacterial composition, alterations in their metabolic activities, or changes in bacterial diversity within the gut is called gut dysbiosis. Dysbiosis has a huge influence on the cause of many diseases such as inflammatory bowel disease, type 1 diabetes, type 2 diabetes, allergies, cancer, obesity, dyslipidemia, and metabolic syndrome [12-18].

Many animal and human studies are showing interest in evaluating a relationship between gut microbial dysbiosis and the development of cardiovascular diseases, mainly atherosclerosis and HTN. Furthermore, the gut microbiome has been recognized as having a key role in obstructive sleep apnea-induced HTN [19]. Proposed mechanisms include the effects of gut microbiota on systemic inflammation, production of pro-atherosclerotic metabolite (trimethylamine *N*-oxide, or TMAO), and short-chain fatty acids (SCFAs) [20,21]. Decreased microbial diversity has been observed in both animal models and human samples of HTN. Population-based human studies have revealed significant associations between microbial metabolites and BP [22].

The exact association between the gut microbiome and HTN is not well studied even if there is a growing interest in the topic. There has been a lack of adequate evidence on the unique alterations in the composition of gut microbiota and metabolic profiles in hypertensives, and whether these changes are a causative factor or a consequence of high BP is still unknown. If the relationship will become more established, the outcome will be greater in the terms of newer therapeutic targets for the prevention of HTN.

This systematic literature review highlights the influence of gut dysbiosis on the pathogenesis of HTN. Further research is required to demonstrate the temporal relationship between dysbiosis and HTN if any.

## Review

Methods

We followed the Preferred Reporting Items for Systematic Reviews and Meta-Analyses (PRISMA), 2020, guidelines while conducting the systematic review [23]. Our main aim is to identify an association between hypertension (HTN) and gut dysbiosis if any.

Literature Search Strategy

For articles that studied an association between intestinal microbial dysbiosis and HTN between 2012 and 2022, we searched the following four databases in detail: PubMed, PubMed Central (PMC), Medline, and ScienceDirect. A systematic search strategy using Medical Subject Headings (MeSH) terms and keywords were developed and used to find the relevant articles for this study. We included the following keywords: “hypertension,” “gut microbiome,” “dysbiosis,” “intestinal dysbiosis,” and “intestinal microbiome.” We also combined the aforementioned keywords and MeSH strategy using the Boolean method to extract all the relevant articles from the databases. The summary of the search strategy for the different databases has been described in Table 1.

**Table 1:** Detailed literature search strategy. PMC, PubMed Central

Database	Strategy	Number of results before inclusion/exclusion criteria	Results after inclusion/exclusion criteria (studies from past 10 years, adult population)
PubMed, PMC, and Medline	(((gutdysbiosis) OR (gut microbiome) OR (intestinal symbiosis) OR (microbiome)) AND ((hypertension)))	1,291	137
	(("Hypertension/adverse effects"[Majr] OR "Hypertension/complications"[Majr] OR "Hypertension/epidemiology"[Majr] OR "Hypertension/microbiology"[Majr] OR "Hypertension/physiopathology"[Majr] )) AND (( "Dysbiosis/diagnosis"[Majr] OR "Dysbiosis/etiology"[Majr] OR "Dysbiosis/pathology"[Majr] OR "Dysbiosis/physiology"[Majr] OR "Dysbiosis/physiopathology"[Majr] ))	13	3
	("Dysbiosis"[Mesh]) AND (( "Hypertension/adverse effects"[Majr] OR "Hypertension/complications"[Majr] OR "Hypertension/epidemiology"[Majr] OR "Hypertension/microbiology"[Majr] OR "Hypertension/physiopathology"[Majr] ))	31	3
	(("Dysbiosis/diagnosis"[Majr] OR "Dysbiosis/etiology"[Majr] OR "Dysbiosis/pathology"[Majr] OR "Dysbiosis/physiology"[Majr] OR "Dysbiosis/physiopathology"[Majr] )) AND ("Hypertension"[Mesh])	26	6
ScienceDirect	(((gut dysbiosis) OR (gut microbiome) OR (intestinal dysbiosis) OR (microbiome)) AND ((hypertension) OR (high blood pressure)) AND ("adults"))	6,506	330

Inclusion Criteria and Exclusion Criteria

Inclusion and exclusion criteria are mentioned in Table 2.

**Table 2:** Detailed inclusion and exclusion criteria.

Inclusion criteria	Exclusion criteria
Papers from last 10 years	Unpublished literature
Papers published in English	Gray literature
Papers focusing on adult population (19+ years)	Papers discussing pediatric population (less than 19 years)
Papers with free full-text articles	
Papers included humans as cases and controls	

Data Extraction and Quality Appraisal of Included Studies

For data extraction, we screened all the included articles first by their title, abstract, and full text to understand their degree of relevance. Consequently, we extracted data including the year of publication, sample size, age of patients, inclusion criteria, results, and conclusions.

For quality appraisal, the Joanna Briggs Institute (JBI, Adelaide, Australia) tool was used. The articles that satisfied >70% of the criteria were finally studied in detail to conduct this study.

Results

Study Selection

**Figure 1:**
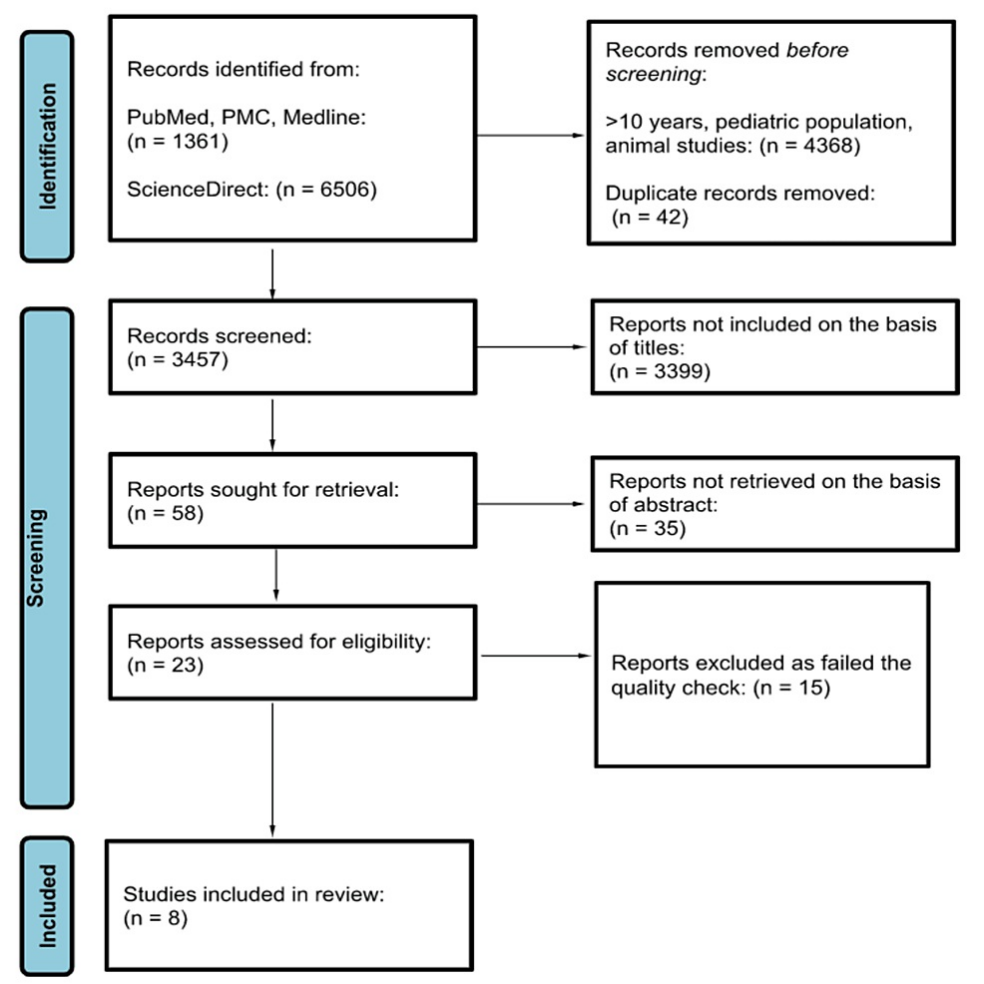
PRISMA flowchart PRISMA, Preferred Reporting Items for Systematic Reviews and Meta-Analyses; PMC, PubMed Central

First, a total of 7,867 articles, from the four databases - PubMed, PubMed Central (PMC), Medline and ScienceDirect, were identified. Consequently, we excluded duplicates, articles older than 10 years, articles focusing on the pediatric population, and animal studies. Then, 3,457 articles remained for further screening based on title, abstracts, and inclusion and exclusion criteria. Among the remaining 23 articles, we excluded the articles that did not fulfill the criteria during quality appraisal using the JBI tool. At last, eight articles were selected to include in this study. All the articles were cross-sectional observational studies. The PRISMA flowchart is shown in Figure 1.

Study Population

The patients (cases) who participated in the study were having systolic BP (SBP) ≥ 140 mmHg and diastolic BP (DBP) ≥ 90 mmHg. Patients were also included from different grades of HTN, including prehypertension (pHTN), grade 1 HTN, and grade 3 HTN.

Gut Composition

Gut microbiota is widely diverse, containing trillions of microorganisms [24]. Most of the studies used the method of 16S ribosomal RNA (*rRNA*) gene amplification and sequencing for evaluating the changes in richness and diversity of gut microbiota in hypertensives. One study also used newly introduced metagenomic sequencing. All these studies concluded that there was a significant difference in the beta diversity (interindividual diversity) in both groups.

The pertinent characteristics of the studies have been discussed in Table 3.

**Table 3:** Characteristics of the studies. BP, blood pressure; SBP, systolic blood pressure; SCFA, short-chain fatty Acid; F/B ratio, Firmicutes/Bacteroidetes ratio; pHTN, prehypertension

Authors	Year of publication	Total number of patients (*n*)	Conclusion/results
Palmu et al. [25]	2020	6,953 (829 for urinary sodium analysis)	Most of the observed changes belonged to the Firmicutes phylum. Analysis was conducted to establish a correlation between sodium intake, gut *Lactobacillus* abundance, and BP.
Sun et al. [22]	2019	529	Associations between within-person and between-person gut microbial community diversity, taxonomic composition, and blood pressure in a diverse population-based cohort of middle-aged adults were seen.
Wang et al. [26]	2021	1,082 (with 1,003 and 434 adults included in the microbiota and metabolomics analysis samples, respectively)	Positive associations of metabolites such as sphingomyelins, acylcarnitines, and cholesterol and potential roles of microbiota and metabolites in BP regulation were seen.
Kim et al. [27]	2018	22	A strong correlation of SBP with gut bacteria and barrier dysfunction existed. Butyrate production is inversely proportional to SBP.
Verhaar et al. [28]	2020	4,672	Substantial differences between gut microbiota composition and high BP in-between ethnic groups were found. A positive correlation was found between BP and fecal SCFA levels.
Mushtaq et al. [29]	2019	80 (50 cases of grade 3 hypertension and 30 controls)	Differences were observed in the bacterial populations, with a critically increased F/B ratio in the patients with grade 3 hypertension.
Li et al. [19]	2017	196 (inclusion of 41 healthy controls, 56 with pHTN, and 99 with primary hypertension)	Significant and minimal differences were found in the structure of gut microbiota between healthy and hypertension and pHTN and hypertension, respectively.
Yan et al. [30]	2017	120 (60 patients with primary hypertension and 60 healthy controls of Chinese origin)	Alterations were found in microbial diversity, genes, and functions of the gut microbiome in hypertensive patients.

Discussion

**Figure 2:**
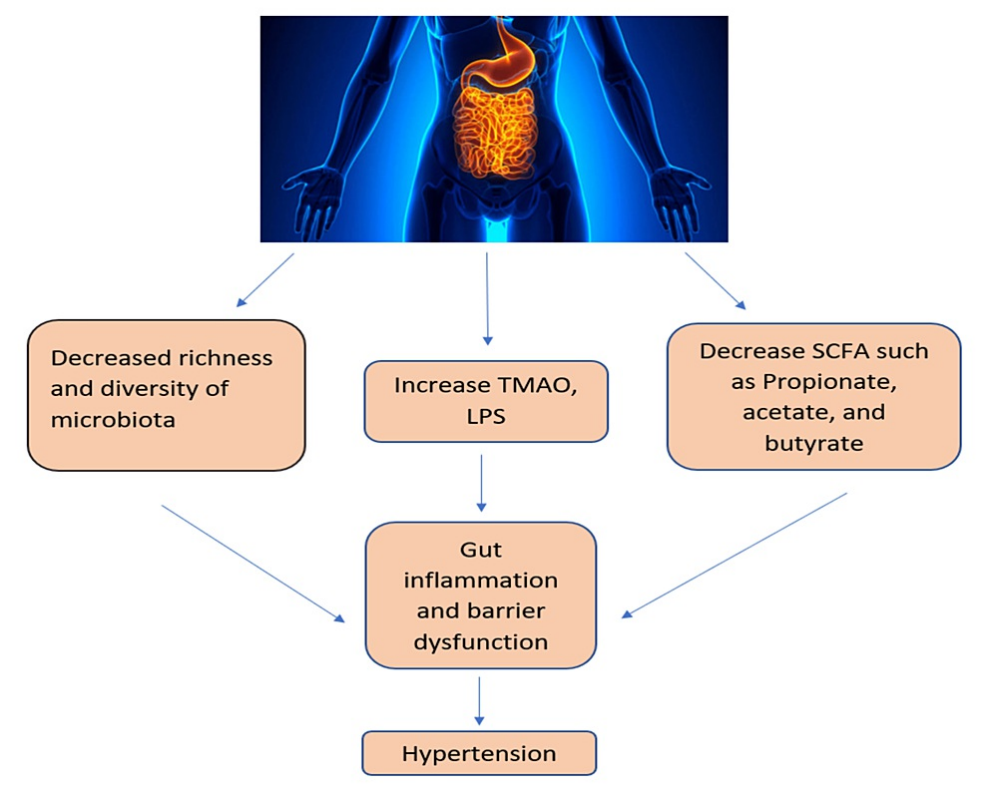
Association between gut dysbiosis and hypertension. TMAO, trimethylamine *N*-oxide; LPS, lipopolysaccharide; SCFA, short-chain fatty acid Figure credits: SSN and NM

It is still challenging to identify and understand the exact mechanism of HTN owing to its complex nature. However, many recent advances are establishing an interplay between gut dysbiosis and HTN. Four phyla dominate the adult human gut: Firmicutes, Bacteroidetes, Actinobacteria, and Proteobacteria. The general idea of how gut dysbiosis and high BP are associated has been shown in Figure 2.

In this study, we have mainly discussed the compositional gut microbiota changes along with the functional changes in genes and metabolome in hypertensive patients to get a detailed picture of the association between the two.

Gut Microbial Richness and Diversity in HTN

A bidirectional communication of gut microbiota exists with the human host and the environment. The human gut is a habitat of trillions of microbiomes, which do have a crucial role in maintaining homeostasis and immunity of the host. Alpha diversity is a diversity observed within a sample (number of species), whereas beta diversity is defined as between-sample diversity (type and abundance of the species). In the following sections, we have discussed altered gut microbiota diversity and richness observed in hypertensive patients. HTN is defined as SBP ≥ 140 mmHg and/or DBP ≥ 90 mmHg [26]. Some basic characteristics of patients, samples, and alterations in the composition have been summarized in Table 4.

**Table 4:** Changes in gut composition seen in hypertensive patients. BP, blood pressure; HTN, hypertension; pHTN, prehypertension

Author	Age (years)	Method used to evaluate bacterial DNA from fecal samples	Changes in gut microbial diversity and richness in HTN patients	Elevated relative abundance in HTN patients	Reduced relative abundance in HTN patients
Palmu et al.[25]	25-74	Shallow shotgun metagenome sequencing	In models adjusted for age and sex, alpha diversity was inversely associated with systolic BP. For age- and sex-adjusted models, all BP indices were significantly associated with beta diversity.	Lactobacillus paracasei	Lactobacillus salivarius
Sun et al. [22]	18-30	MoBio PowerSoil kit (Qiagen, Hilden, Germany) Sequencing (of hypervariable V3 and V4 region of *16S rRNA*) on the Illumina MiSeq (San Diego, CA, USA) platform (2×300)	Alpha diversity was inversely associated Significant differences in beta diversity	*Catabacter* and *Robinsoleilla*	*Sporobacter* and *Anaerovorax*
Wang et al. [26]	30-69	TIANGEN (Beijing, China) DNA extraction kits Sequencing for *16S rRNA* targeting the V4 hypervariable region performed on the Illumina MiSeq PE250 platform	Differences in beta diversity	*Rothia*, *Serratia*, Enterobacteriaceae, Leuconostocaceae, and *Fusobacterium*	*Coprococcus*, *Adlercreutzia*, *Eggerthella*, and *Raistonia*
Kim et al. [27]	>18	With PowerFecal DNA extraction kit (Qiagen)	-	*Ruminococcus torques*, *Eubacterium siraeum*, and *Alistipes finegoldii*	Bacteroides thetaiotaomicron
Verhaar et al. [28]	18-70	Sequencing the V4 region of the *16S rRNA* gene on Illumina MiSeq	Alpha diversity difference stronger with young female (Dutch)	*Streptococcus* spp. and *Klebsiella* spp.	*Roseburia* spp., *Clostridium sensu stricto* spp., *Roseburia hominis*, *Romboutsia* spp., and Ruminococcaceae
Mushtaq et al. [29]	40-75	*16S rRNA* gene amplification using the QIAamp MiniStool kit (Qiagen)	Alpha diversity significantly higher in the hypertension group	Veillonellaceae family, *Prevotella*, *Megasphaera*, *Parasutterella*, *Escherichia*, and *Shigella*	*Faecalibacterium prausnitzii* and *Bacteroides uniformis*
Li et al. [19]	-	TIANGEN kit	Inversely related alpha diversity in both pHTN and HTN groups	*Prevotella* and *Klebsiella*	*Faecalibacterium*, *Oscillibacter*, *Roseburia*, *Bifidobacterium*, *Coprococcus*, and *Butyrivibrio*
Yan et al. [30]	-	QIAamp DNA mini kit (Qiagen)	Alpha diversity inversely associated in hypertensives	*Klebsiella*, *Streptococcus*, and *Parabacteroides* (*P. merdae*)	*F. prausnitzii*, Roseburia spp., and Synergistetes

Palmu et al. studied the alpha and beta diversities, and they found a significant change in beta diversity with SBP and DBP, but an inverse association of alpha diversity with SBP in age- and sex-adjusted studies [25]. In multi-variable-adjusted models, beta diversity with DBP was only identified. Among the observed changes in taxonomic composition, most of them belonged to the Firmicutes phylum. To offer novel insights, a more focused analysis was conducted to establish a correlation between sodium intake, gut *Lactobacillus* abundance, and BP. They highlighted the potential mechanism of a diet contributing to change the gut microbiome, resulting in high BP by observing the abundance of *Lactobacillus paracasei* with decreased urinary sodium excretion and *Lactobacillus salivarius* with increased urinary sodium excretion. It cannot be ignored that the method used for sequencing and labeling DNA (shallow shotgun metagenome) is a new field with reported limitations.

Sun et al. found that the richness in various taxonomic groups was inversely related to high BP. Inverse cross-sectional relation of alpha diversity with systolic BP was observed in Coronary Artery Risk Development in Young Adults (CARDIA) participants [22]. They observed elevated representation of *Catabacter* and *Robinsoleilla* and decreased representation of *Sporobacter* and *Anaerovorax*. However, findings were found to be sensitive after adjustment of covariants such as body mass index (BMI). The major limitation of the study was that one-third of the patients included were taking antihypertensives.

To minimize the effect of antihypertensives on the results, Wang et al. conducted a study. They took participants from China Health and Nutrition Survey (CHNS) due to the presence of the highest number of undiagnosed and untreated hypertensive patients in addition to the presence of high prevalence of HTN in China. Hence, according to them, China might be the ideal place to study HTN [26]. Additionally, a few patients who were using medication to control BP were excluded from the study. The essential characteristics were a positive correlation of HTN with *Rothia*, *Serratia*, Enterobacteriaceae, Leuconostocaceae, and *Fusobacterium*, while a negative correlation with *Coprococcus*, *Adlercreutzia*, *Eggerthella*, and *Raistonia*.

The focus of the study by Verhaar et al. was to recognize an association and to find substantial differences between gut microbiota composition with high BP between ethnic groups within the population-based HEalthy Life In an Urban Setting (HELIUS) cohort [28]. The study showed the most significant alpha diversity in young female Dutch hypertensives among other ethnic groups. They noted the elevated abundance of *Streptococcus* spp. and *Klebsiella* spp. and a reduced abundance of *Roseburia* spp., *Clostridium sensu stricto* spp., *Roseburia hominis*, *Romboutsia* spp., and Ruminococcaceae. SCFAs are end products of intestinal fermentation done by gut microbiota, which are otherwise indigestible dietary products. Some examples of SCFA are propionate, acetate, and butyrate. Butyrate is majorly used by colonocytes, while acetate and propionate are consumed by the liver. SCFAs regulate gut inflammation and metabolism by functioning as an important colonocytes energy source and signaling molecules, indicating a low level of SCFA production in gut microbiota may act as a risk factor for multiple metabolic syndromes, including HTN. *G-protein coupled* free fatty acid receptors (FFARs) are present in the renal arteries, resulting in arterial vasodilation when attached with SCFAs. The positive correlation found between BP and fecal SCFA levels in the study was conflicting, with the negative correlation found between BP and SCFA-producing bacteria. With further research, researchers understood that they were measuring fecal SCFA levels as a proxy for intestinal SCFA production, which could be not reflecting the exact level of intestinal SCFA production. The measured fecal SCFA might reflect the amount after subtracting SCFA absorption. Consequently, they hypothesized that higher microbial SCFA production increases intestinal SCFA absorption, resulting in lower levels of SCFAs excretion in feces.

Kim et al. observed that bacterial taxa such as *Ruminococcus torques*, *Eubacterium siraeum*, and *Alistipes finegoldii* were increased in patients [27]. *R. torques* have been studied to participate in mucus degradation and eventually in gut barrier dysfunction. *E. siraeum* and *A. finegoldii* are known to trigger intestinal inflammation. In contrast, a decreased number of *Bacteroides thetaiotaomicron* was observed, which boosts the immunity of the host by strengthening the mucosal barrier. Depletion of *Eubacterium rectale*, one of the most significant butyrate-producing bacteria of the human gut, was observed. The study elucidated that butyrate production is inversely proportional to SBP. The finding was additionally validated by plasma butyrate measurements that were on the lower side. Furthermore, *Rosebria* and *Eubacterium* were on the higher side in the healthy people, which are the major butyrate-producing genera. Apart from being a small sample-size study, participants were divided without considering possible confounding factors such as diet, culture, demographics, and race.

Another study by Mushtaq et al. was unique as they took patients with grade 3 HTN and compared with the healthy controls. Firmicutes and Bacteroidetes are two of the four phyla that dominate the human gut flora. The Firmicutes/Bacteroidetes ratio (F/B ratio) was used to characterize gut dysbiosis. An increased ratio in the HTN group compared with the control group was identified. An increased F/B ratio could be due to an increase in Firmicutes or a decrease in Bacteroidetes [29]. An increase in abundance was noted of the Veillonellaceae at the family level and *Prevotella*, *Megasphaera*, *Parasutterella*, and *Escherichia‑Shigella* at the genus level compared with the control group. Veillonellaceae produces acetate and propionate in high amounts. *Prevotella copri* promotes inflammation by expressing a superoxide reductase. They did not find any major differences among *Klebsiella* spp., *Streptococcus* spp., and *Parabacteroides merdae*.

Yan et al. reported frequent distribution of them in hypertensives. Using the Metagenome-wide Association Studies (MGWAS) method, 31 Metagenome Linkage Groups (MLGs) were found to be higher in patients while 37 in controls [30]. In controls, MLGs of *Roseburia* and *Faecalibacterium prausnitzii* were on a higher side. They hypothesized a gross *dose-response* relationship between the abundance of HTN/control-enriched MLGs and the severity of HTN. At the phylum level, patients had higher levels of Proteobacteria but fewer Actinobacteria. At the genus level, *Klebsiella*, *Clostridium*, *Streptococcus*, *Parabacteroides*, *Eggerthella*, and *Salmonella* were frequently distributed in the hypertensives, while *Faecalibacterium*, *Roseburia*, and *Synergistetes* were found to be higher in the control group. *F. prausnitzii* and *Roseburia* are the major SCFA producers in the human colon, which might lead to the depletion of SCFA-producing enzymes in hypertensives. The major limitation was that 35% of patients had taken antihypertensive drugs or specific nutritious supplements.

A most unique part of the study conducted by Li et al. is that they included three groups to study the association: hypertensives, prehypertensives (pHTN), and healthy controls. On top of that, all the subjects included in the HTN group were newly diagnosed hypertensives. Thus, patients were not exposed to antihypertensive medication. According to them, the rate of acquiring new genes in controls surpassed the rate for the same in disease samples, which directly indicated a low richness of genes in hypertensives [19]. *Prevotella* and *Klebsiella* were found to be increased in HTN. Inflammation due to an immune response triggered by lipopolysaccharide (LPS) is the fundamental feature of gram-negative bacteria’s pathogenesis, such as *Prevotella* and *Klebsiella*. In addition to that, *Prevotella* is also associated with stearic acid, an important metabolite in HTN. *Faecalibacterium*, *Oscillibacter*, *Roseburia*, *Bifidobacterium*, *Coprococcus*, and *Butyrivibrio* were enriched in controls. *Faecalibacterium* and *Roseburia* in the intestines are very important for butyric acid production. Moreover, *Bifidobacterium* is necessary probiotics for gut microbial homeostasis, LPS reduction, and gut barrier. In pHTN also, they observed somewhat similar alterations in the bacterial diversity, enterotype, composition, and metabolic functions. After observing a minute difference in the characteristic of gut microbiota between pHTN and HTN, they recognized that pHTN is, in fact, a state in which gut dysbiosis has already occurred and not only a transitional period between normotensive and hypertensive status. As a result, they emphasized intervening early in pHTN patients instead of neglecting pHTN populations. However, it was a small sample size study.​​​​​​​

Functional and Metabolomic Alterations in HTN

A study of changes in the functional expressions of the genes and the metabolome may fill the gap and allow us to understand the communication between gut microbiota and elevated BP in more detail. The metabolome is like a snapshot of different metabolic pathways by which we can identify new biomarkers responsible for elevation of the blood pressure. So along with studying compositional changes, it is beneficial to study these changes as well.

Wang et al. found positive associations of metabolites such as sphingomyelins, acylcarnitines, and cholesterol with both SBP and DBP [26]. Ceramide, a precursor of sphingolipids, has been shown to be detrimental to the heart in many ways. One of them is to impair vasodilation. When fatty acids surpass the energy requirement or storage room of a cell, an overabundance of sphingolipids takes place. They inferred sphingolipids as a potential biomarker for cardiovascular diseases in humans. Acylcarnitines, byproducts of incomplete β-oxidation, can accumulate in the blood when fatty acids are in excess for the oxidation process. They stimulate proinflammatory pathways involving nuclear factor-kappa B, which eventually results in high BP.

Another study that reflected on metabolomic changes was done by Kim et al. They evaluated impaired gut barrier function in the high BP cohort by measuring intestinal fatty acid binding protein (I-FABP), LPS, and soluble form of zonulin in plasma, which are biomarkers for gut epithelial health [27]. Zonulin is a protein present in the gut epithelial tight junction, which helps in the regulation of the junction. Moreover, they observed a significant increase in Th17 cells expressing CD161 and CCR6/integrin β7 in the HTN cohort, which are markers for gut inflammation. A constellation of these changes could be suggesting the interrelation between decreased gut barrier function and increased inflammation in patients with HTN.

From a metabolomic perspective, Yan et al. revealed that HTN-enriched taxonomic groups were involved more in membrane transport, LPS biosynthesis, and steroid degradation. Healthy controls demonstrated microbiota involved in the metabolism of *other amino acids*, cofactors, and vitamins (including folate biosynthesis and metabolism, riboflavin metabolism, and ubiquinone biosynthesis) [30]. Furthermore, the gut microbial enzymes, which are involved in trimethylamine *N*-oxide (TMAO) production, were enriched and the SCFA-producing enzymes were depleted in hypertensives. The choline utilization (*cutC*) gene, a gene that degrades the choline into trimethylamine, was identified in a variety of human gut commensals. Functional analysis also demonstrated the abundance of *cutC* genes in the gut microbiota of the hypertensives. Notably, several genera such as *Klebsiella* and *Streptococcus*, which were found to be in higher numbers, are choline degraders. These findings suggested that choline intake and TMAO production by gut microbiota could contribute to the pathogenesis of HTN.

Li et al. observed depletion in genes associated with biosynthesis and transport of amino acids, such as lysine, histidine, leucine, and serine, which are essential for human health in HTN patients [19]. An analysis of bacterial gene functions suggested that overproduction of LPS by gut bacteria could contribute to HTN pathogenesis; on the other hand, biosynthesis of amino acids, fatty acid utilization, and purine metabolism by gut bacteria might prevent the development of HTN.

Palmu et al. suggested the possibility of an association between microbiome-driven processes related to lipid metabolism, gluconeogenesis, and xenobiotic metabolism with blood pressure [25]. However, they proposed further validation of the hypothesis of functional changes by experimental studies with detailed sequencing.

Limitations

The sampling method determines results. Ideally, mucosal biopsy should be used for studying microbiota composition and diversity instead of fecal sampling because it is only a proxy. Mucosal microbiota can be different from stool microbiota. Mucosal sampling has its own limitations too, such as disruption of the biofilm while taking the biopsy and also the impact of bowel preparation before a colonoscopy. The gut microbiota is affected by many factors, including age, diet, medications, exercise, and other coexisting diseases such as gut malignancy. Many studies did not take them into consideration or only took one factor into consideration. Many articles are present in the Chinese language on a similar subject, but due to the language barrier, we could not include them in this study. No clinical trial studies have been included in this study. Thus, large-scale, multicenter longitudinal studies are required for a better understanding of the association between gut dysbiosis and HTN, if any.

## Conclusions

This study demonstrates a consistent relationship between high BP and gut microbiota ecology. Continuous cross-talk between the two signifies the potential mechanism of HTN pathogenesis. The richness and composition also depend on the diet, age, ethnicity, and severity of HTN. Metabolomic changes seen in hypertensives alter the previously well-maintained homeostasis and promote a proinflammatory state. Metabolites present in plasma and feces such as I-FABP, LPS, zonulin, sphingomyelins, acylcarnitines, and TMAO require validation by further studies to explore their potential as biomarkers. To establish a further causal relationship, more studies are warranted.
